# *PrMYB5* activates anthocyanin biosynthetic *PrDFR* to promote the distinct pigmentation pattern in the petal of *Paeonia rockii*

**DOI:** 10.3389/fpls.2022.955590

**Published:** 2022-08-03

**Authors:** Qianqian Shi, Meng Yuan, Shu Wang, Xiaoning Luo, Sha Luo, Yaqi Fu, Xiang Li, Yanlong Zhang, Long Li

**Affiliations:** ^1^College of Landscape Architecture and Art, Northwest A&F University, Yangling, China; ^2^Co-Innovation Center for Sustainable Forestry in Southern China, Nanjing Forestry University, Nanjing, China; ^3^Bamboo Research Institute, Nanjing Forestry University, Nanjing, China

**Keywords:** *Paeonia rockii*, *PrMYB5*, *PrDFR*, anthocyanin biosynthesis, petal spot

## Abstract

*Paeonia rockii* is well-known for its distinctive large dark-purple spot at the white petal base and has been considered to be the main genetic source of spotted tree peony cultivars. In this study, the petal base and petal background of *Paeonia ostii* (pure white petals without any spot), *P. rockii*, and other three tree peony cultivars were sampled at four blooming stages from the small bell-like bud stage to the initial blooming stage. There is a distinct difference between the pigmentation processes of spots and petal backgrounds; the spot pigmentation was about 10 days earlier than the petal background. Moreover, the cyanin and peonidin type anthocyanin accumulation at the petal base mainly contributed to the petal spot formation. Then, we identified a C1 subgroup R2R3-MYB transcription factor, *PrMYB5*, predominantly transcribing at the petal base. This is extremely consistent with *PrDFR* and *PrANS* expression, the contents of anthocyanins, and spot formation. Furthermore, *PrMYB5* could bind to and activate the promoter of *PrDFR* in yeast one-hybrid and dual-luciferase assays, which was further verified in overexpression of *PrMYB5* in tobacco and *PrMYB5*-silenced petals of *P. rockii* by comparing the color change, anthocyanin contents, and gene expression. In summary, these results shed light on the mechanism of petal spot formation in *P. rockii* and speed up the molecular breeding process of tree peony cultivars with novel spot pigmentation patterns.

## Introduction

Petal spot is a kind of regular variegated pattern of flower color, which exists in angiosperm, such as *Nomocharis meleagrina, Torenia fournieri, Sparaxia elegans, Dendrobium nobile*, and *Paeonia suffruticosa* (Gao et al., 2015; Zhang et al., 2015; Shi et al., 2017; Su et al., 2017; Gu et al., 2019). It not only imparts ornamental and commercial values but also affects the preferences of pollinators and promotes the success of pollination (Davies et al., 2012; Glover et al., 2013), so the molecular mechanism of petal spot formation has always been a research hotspot.

The formation of petal spots is determined by the specific spatiotemporal transcription of genes in the anthocyanin biosynthesis pathway (ABP), resulting in specific anthocyanins accumulation in defined petal regions (Suzuki et al., 2016). In ABP, chalcone synthase (*CHS*), chalcone isomerase (*CHI*), flavanoid 3'-hydroxylase (*F3'H*), flavanone 3-hydroxylase (*F3H*), dihydroflavonol 4-reductase (*DFR*), anthocyanidin synthase (*ANS*), and flavonoid glucosyltransferase (*UFGT*) are mainly controlled by MYB transcription factor (TF), basic helix–loop–helix (bHLH), and WD40 protein in different plants (Cao et al., 2020; Yan et al., 2021). To date, the spatio-temporal regulation mechanisms involved in petal spot formation have been explored in many ornamental plants, including *Antirrhinum* spp.*, Petunia hybrid, Lilium* spp.*, Mimulus* spp.*, Clarkia gracilis*, and *Phalaenopsis* spp. (Schwinn et al., 2006; Albert et al., 2011; Shang et al., 2011; Yamagishi, 2011, 2020; Yamagishi et al., 2010; Glover et al., 2013; Yuan et al., 2014; Hsu et al., 2015; Martins et al., 2017). In the same way, *CgMYB1* activated *CgDFR2* and *CgANS* to produce petal spots in *C. gracilis* (Martins et al., 2017). However, the specific regulation mechanism of petal spot is rarely studied in woody ornamental plants, especially wild tree peony species.

Tree peony is one of the most famous traditional flowers in China and is popular all over the world for its large, colorful, and distinct flowers. At present, there are more than 2,000 cultivars worldwide with various petal pigmentation patterns, such as spots, stripes, and more complex designs (Zhou et al., 2014). *Paeonia rockii* is the most important wild tree peony species in Northwest China and has a large dark-purple spot at the white petal base, which has been confirmed as a main genetic source of spot pigmentation in tree peony (Wang et al., 2000; Shi et al., 2017). *Paeonia ostii* has pure white petals without any spots, and other tree peony cultivars of *P. suffruticosa*, “Lanhudie” (LHD), “Mochi Jinhui” (MCJH), and {High Noon” (HN), possess different degrees of purple spots on differentially colored petal backgrounds, which can be considered as the control group for the spot pigmentation study of *P. rockii*. Therefore, it is urgent to explore the molecular mechanism of petal spot pigmentation of *P. rockii* to accelerate the molecular breeding process of a variegated tree peony in China. Previous research demonstrated that the spot formation at the petal base of tree peony was primarily attributed to the spatial biosynthesis of cyanidin (Cy) and peonidin (Pn) anthocyanins (Zhang et al., 2015; Shi et al., 2017). The anthocyanin structural genes and anthocyanin regulatory MYBs have been identified in tree peony cultivars, including *PsMYB114L, PsMYB12L, PsMYB12, PsMYB111, PsMYB4, PsMYB57*, and *PsMYB58* (Gu et al., 2019; Zhang et al., 2019, 2020a,b; Luo et al., 2021). Among them, *PsMYB12* directly activated the expression of *PsCHS* by forming an MBW regulatory complex with bHLH and WD40 proteins, which were specific to the petal blotch in tree peony cultivar “Qing Hai Hu Yin Bo” (Gu et al., 2019). However, whether and how other MYBs regulated the petal spot formation in *P. rockii* are almost unknown.

Previously comparative transcriptome analyses of *P. rockii* and *P. ostii* flowers suggested that *CHS, DFR, ANS*, glutathione S-transferase (*GST*), and two R2R3-MYB TFs might be related to petal spot formation in *P. rockii* (Shi et al., 2017). In the present study, we investigated one anthocyanin regulatory R2R3-MYB TF *PrMYB5* in *P. rockii* and analyzed the expression patterns at different parts of petals during flower opening of *P. rockii, P. ostii*, and other three tree peony cultivars. Furthermore, we conducted gene overexpression in tobacco plants, virus-induced gene silencing (VIGS), and RNA *in situ* hybridization to verify that *PrMYB5* can individually regulate the distinct petal pigmentation in *P. rockii* by activating *PrDFR* expression, resulting in anthocyanin accumulation. The results aim to provide valuable resources and theoretical supports for molecular breeding of variegation cultivars in tree peony and supply references for the studies on the spot formation mechanism in other woody plants.

## Materials and methods

### Sample preparation

**Figure 1:**
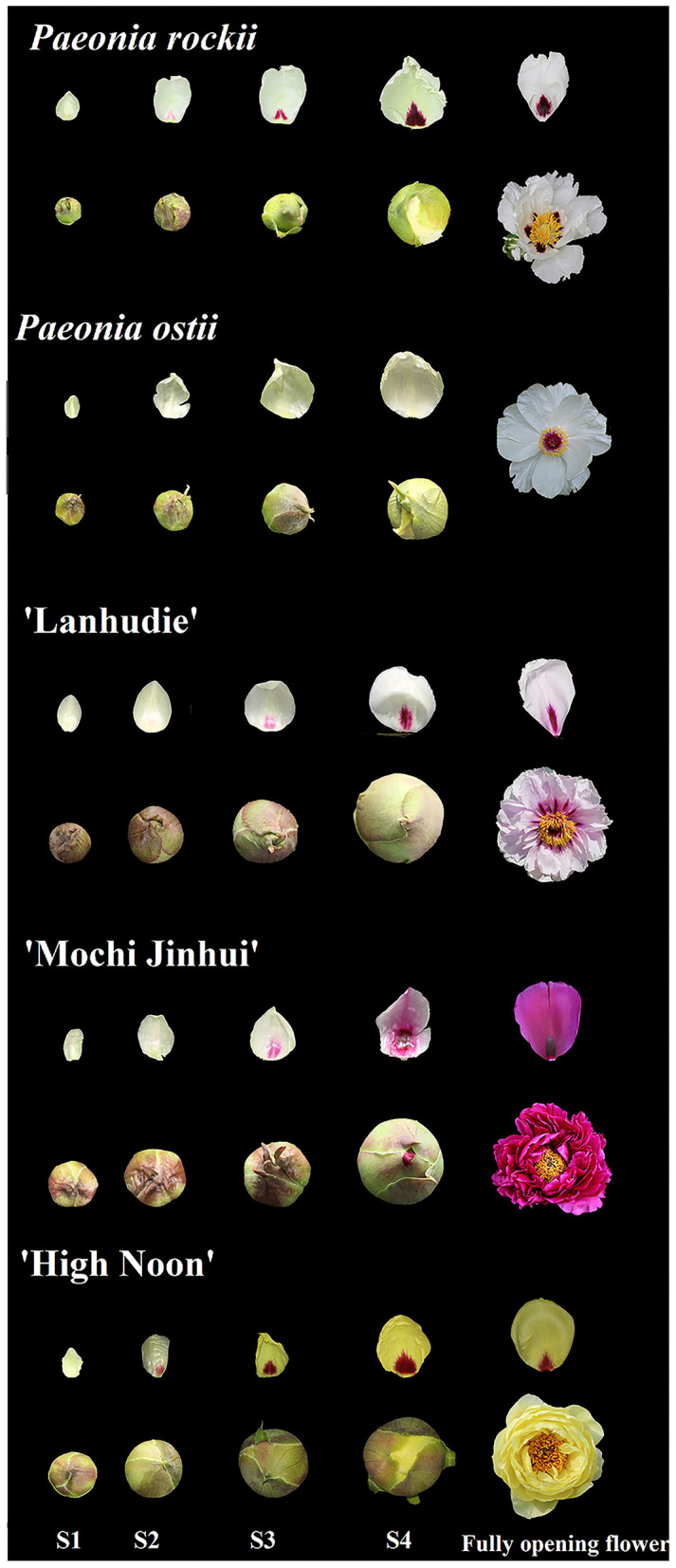
Flower phenotypes of *Paeonia rockii, P. ostii, P. suffruticosa* “Lanhudie,” “Mochi Jinhui,” and “High Noon” at four different opening stages. S1: stage 1, small bell-like bud stage when the petals were mainly yellow-green; S2: stage 2, large bell-like bud stage when the petal bases turn obvious color with the yellow-green background; S3: stage 3, bell-like bud extending stage when spots get bigger and darker; S4: stage 4, initiating blooming stage when spots are completely formed.

The plants of *P. rockii, P. ostii*, and three tree peony cultivars *P. suffruticosa*, “Lanhudie,” “Mochi Jinhui,” and “High Noon,” were grown in the germplasm repository of the Northwest A&F University, Shaanxi, China. Petal backgrounds and petal bases at four different blooming stages were sampled separately from March to April, 2018 (Figure 1). The four blooming stages include: stage 1 (S1): a small bell-like bud stage when the petals are mainly yellow-green; stage 2 (S2): a large bell-like bud stage when the petal bases turn obvious color with the yellow-green background; stage 3 (S3): a bell-like bud extending stage when spots get bigger and darker; and stage 4 (S4): an initiating blooming stage when spots are completely formed (Figure 1). Tobacco plants (*Nicotiana tabacum* L. cv. K326) were grown in a conservatory under 16 h/8 h light photoperiod at 25°C. The color indices (L^*^, a^*^, b^*^, *C*, and *h*) were measured in the fresh petals at four stages using a colorimeter (CR-400, Konica Minolta, Osaka, Japan). The other materials were immediately frozen in liquid nitrogen and then stored at −80°C.

### HPLC analysis

Flavonoids were detected in tree peony petals and tobacco petals using the high-performance liquid chromatography (HPLC) method, as previously reported (Luo et al., 2021). First, each sample was ground into powder in liquid nitrogen. Then, 1 mg of petal powder was extracted with 2 ml methanol-formic acid (99:1, V/V) at 4°C for 24 h in the dark. After ultrasonic-assisted extraction for 30 min and centrifugation at 10,000 rpm for 10 min, liquid supernatant was gathered and filtered using a 0.22 μm nylon microporous membrane. Flavonoid analysis was done as follows: eluent A: 0.04% formic acid; eluent B: acetonitrile. Elution procedure: 1–40 min: 5–40% B; 40–45 min, 40–100% B; 45–55 min, 100%B; 55–60 min, 100–5% B. Flow rate: 0.5 mL ∙min^−1^, column temperature: 40°C, injection volume: 10 μL. We used Cy3G, Cy3G5G, Pg3G, Pg3G5G, Pn3G, Pn3G5G, Ap, Ch, Is, Km, Lu, and Qu from the Shanghai Yuanye Bio-Technology Co., Ltd (Shanghai, China) as standards. All assays were conducted with three biological replicates.

### Isolation of full-length CDNA, sequence alignment, and phylogenetic tree analysis

Total RNA was extracted from 100 mg petal base of *P. rockii* at S4 using the Quick RNA Isolation Kit (Bioteke Corporation, Beijing, China). After RNA purity and integrity were checked, full-length cDNAs of *PrMYB5* were cloned using the SMARTer RACE 5'/3' Kit (TAKARA Corporation, Beijing, China) according to the manufacturer's protocols. The primers are listed in Supplementary Table 1.

The multiple sequence alignment and phylogenetic tree of PrMYB5 and some anthocyanin biosynthesis and spot formation related R2R3-MYB proteins from tree peony and other plants were performed using the DNAMAN6.0.3.99 software and the MEGA 6.0 software using the neighbor-joining method, respectively.

### Subcellular localization

The *PrMYB5* ORF was cloned into the pC2300-GFP vector with the *KpnI*and *SalI* restriction sites using In-Fusion Cloning and then transformed into the onion epidermal cells through particle bombardment experiment. After onion tissues were cultured at 25°C for 24 h in the dark, we used a laser confocal microscope (Leica TCS SP2) to observe the fluorescence. All primers are listed in Supplementary Table 1.

### RNA *in situ* hybridization

RNA *in situ* hybridization was conducted according to the previously reported method (Li et al., 2018). The stage 4 petal background and petal base of *P. rockii* were fixed in 4% paraformaldehyde at 4°C for 24 h. After being dehydrated through a graded ethanol series and then embedded in paraffin, the petals were sectioned into 10 μm thick and followed by dewaxing with xylene, rehydrating through an ethanol series, pretreating with proteinase K (1μg mL^−1^) in 1×PBS at 37°C for 30 min, prehybriding and hybriding followed previous protocols: two times of 1× SSC at 45°C for 20 min and two times of 0.5× SSC at 42°C for 15 min (Hsu et al., 2015; Li et al., 2018). The antisense and sense probes of *PrMYB5* were synthesized using gene-specific primers with T7 and SP6 RNA polymerase-binding sites, which are shown in Supplementary Table 1.

### qRT-PCR analysis

Total RNAs were extracted and reverse transcribed into first-strand cDNA for real-time quantitative PCR (qRT-PCR) using the PrimeScript^TM^ RT Master MIX reverse transcription kit (TaKaRa, Beijing, China). qRT-PCR was conducted using SYBR^®^ Premix Ex Taq^TM^ II (Perfect Real Time) Kit (TaKaRa, Beijing, China) on a Bio-Rad CFX96^TM^ Real-Time system (Bio-Rad, Hercules, CA, USA) according to the reaction conditions mentioned previously (Luo et al., 2021). The transcription levels of genes were normalized to the *PsUbiquitin* expression level and calibrated to the expression level in *P. ostii* petal background at S1. Three biological replicates and technical replicates were performed for each reaction. All primers are listed in Supplementary Table 1.

### Interaction network analysis

Based on the transcripts of *PrMYB5* and the structural genes and flavonoid components, a network was established using the R package (https:// www. r- project.org) with a coefficient of *R* ≥ 0.5 or *R* ≤ −0.5. The hub genes were determined to be co-expressed genes with strong interconnections. Finally, the network was visualized using Cytoscape (v.3.1.0; Shannon et al., 2013).

### *Generation of PrMYB5*-overexpressing tobacco plants

The ORF of *PrMYB5* was cloned into the pCAMBIA1300 vector, and the constructs were transformed into tobacco using the previously described method (Horsch and Klee, 1986). After T0 generation transgenic tobacco was obtained, we screened overexpressed-*PrMYB5* transgenic lines (OE-PrMYB5) using PCR and collected seeds until the T2 generation OE-PrMYB5 plants were obtained. Finally, three T2 generation OE-PrMYB5 lines were selected to detect color indices, quantify flavonoid levels, and qRT-PCR verification as described above. All primers are listed in Supplementary Table 1.

### Transient silencing of *PrMYB5* in petals of *P. rockii* by virus-induced gene silencing (VIGS)

According to the previous VIGS approach (Luo et al., 2022), about 200 bp fragment at the 3' end of *PrMYB5* ORF was cloned into the TRV2 vector digested with KpnI and XhoI. The TRV2-*PrMYB5* construct, TRV1, and TRV2 empty vectors were transferred into *Agrobacterium* strain GV3101. Then, the mixtures with *Agrobacterium* containing TRV1 and that containing TRV2, TRV2-*PrMYB5* at a ratio of 1:1 were cultured for 4 h at 25°C in the dark, and then infected fresh petal discs with 1.2 cm diameter from the center of petal background and petal base of *P. rockii* at S4 through vacuum infiltration (Luo et al., 2022). After vacuuming for 2 days, petal discs were washed with deionized water and collected for further analysis. All primers are listed in Supplementary Table 1.

### Cloning and sequence analysis of *PrDFR* and *PrANS* promoter region

The promoters of *PrDFR* and *PrANS* were cloned from the S4 petal genomic DNA of *P. rockii* using the Genome Walking Kit (TAKARA, Beijing, China). Based on the mRNA sequences of *PrDFR* and *PrANS*, three reverse primers, SP1, SP2, and SP3, were used in three rounds of PCR for *PrDFR* and *PrANS* promoters cloning, respectively (Supplementary Table 1). The forward primer was AP4 in all PCR reactions, which was supplied in the kit. *cis*-elements analysis was detected using the online software PlantCARE (http://bioinformatics.psb.ugent.be/webtools/plantcare/html/; Lescot et al., 2002).

### Yeast one-hybrid assay (Y1H)

First, the *PrMYB5* ORF was inserted into the pGADT7 vector (*SmaI* and *SacI* restriction sites) to obtain pGADT7-*PrMYB5*. The *PrDFRpro* and *PrANSpro* were cloned into the pHIS2 vector with *EcoR I* and *Sac I* restriction sites. Then, all constructs co-transformed pairwise into yeast strain AfHY187 according to the lithium acetate method (Zhang et al., 2018). All primers used in Y1H are shown in Supplementary Table 1.

### Dual-luciferase assays

The *PrDFRpro* and *PrANSpro* were cloned into the pGreenII 0800-LUC vector between *Sal I* and *Hind III* restriction sites to obtain *PrANSpro*-pGreenII 0800-LUC and *PrDFRpro*-pGreenII 0800-LUC constructs, respectively. In the meantime, the ORF of *PrMYB5* was inserted into the pGreenII 62-SK vector using *BamH I* and *EcoR I* restriction sites to obtain the *PrMYB5*-pGreenII 62-SK construct. All primers are listed in Supplementary Table 1. After transforming into *Agrobacterium* strain GV3101, the mixtures of cultures (*PrMYB5*-SK+*PrANS*-0800, *PrMYB5*-SK+*PrDFR*-0800) were infiltrated into *N. benthamiana* leaves with needleless syringes (Zhang et al., 2018). After 72 h infiltration, firefly luciferase (LUC) and renilla luciferase (REN) were assayed. The LUC to REN ratio was calculated to measure the binding activity of *PrMYB5* to the promoters. Each combination was conducted in three biological replicates and three technical replicates.

## Results

### Petal spot pigmentation during flower opening

Petal basal spots began to emerge at the small bell-like bud stage (S1) when petals were not colored and fully formed until the blooming stage (S4). However, petals began to color from the bell-like bud extending stage (S3) after 10 days (Figure 1). The same phenomenon also appeared in other tree peony cultivars with different petal spot patterns (Figure 1).

Then, the color indices of petal tissues of *P. rockii* (PR), *P. ostii* (PO), “Lanhudie” (LHD), “Mochi Jinhui” (MCJH), and “High Noon” (HN) were measured at S1–S4 (Supplementary Figure 1). According to the Royal Horticultural Society Color Chart (RHSCC), the background color of PR at S1–S4 was the same as that of PO petals. The petal base colors of PR and PO were green at S1, and then the petal base color of PR varied greatly from green to dark purple at S2–S4 (Supplementary Table 2). At the petal bases of PR, a^*^ (redness) value raised gradually from S1 to S4, while b^*^ (yellowness) value decreased; the L^*^ (lightness) values peaked at S2, and then subsequently declined from S3 to S4 (Supplementary Figure 1A); *h* (hue angle) and C^*^ (chroma) decreased from S1 to S3, and then increased at S4; *h*-value at S4 was ~ 0° (360°), consistent with the dark-purple color (Supplementary Figure 1A). However, color indices of petal bases in PO changed slightly during flower opening, which were similar to those of petal background in PO and PR (Supplementary Figure 1B). Furthermore, the color of petal spots at the S4 stage in LHD, MCJH, and HN were moderate purplish-red, dark purple, and strong red, respectively (Supplementary Table 2). Similarly, L^*^ and b^*^ values declined, and conversely, a^*^ value increased from S1 to S4 at the petal base of LHD, MCJH, and HN (Supplementary Figures 1C–E). The values of *h* and C^*^ were all consistent with their petal base colors (Supplementary Figures 1C–E).

**Figure 2:**
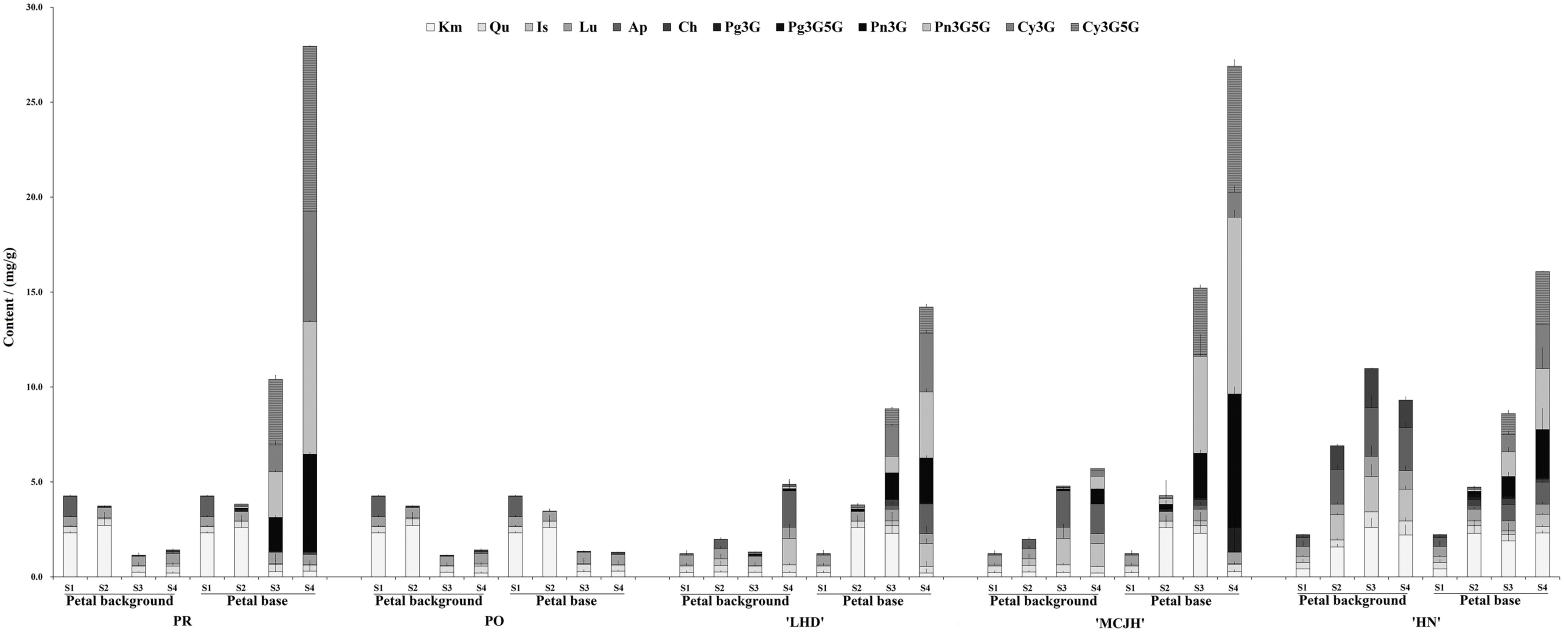
The contents of flavonoids in petal background and base of *P. rockii* (PR), *P. ostii* (PO), and three tree peony cultivars “Lanhudie” (LHD), “Mochi Jinhui” (MCJH), and “High Noon” (HN) during petal spot formation. S1–S4 represent four different blooming stages. Cy3G, cyanidin 3-O-glucoside; Cy3G5G, cyanidin 3, 5-O-glucoside; Pg3G, pelargonidin 3-O-glucoside; Pg3G5G, pelargonidin 3, 5-O-glucoside; Pn3G, peonidin 3-O-glucoside; Pn3G5G, peonidin 3, 5-O-glucoside; Km, kaempferol; Qu, quercetin; Lu, luteolin; Is, isorhamnetin; Ap, apigenin; Ch, chrysoeriol.

Significant differences in flavonoid compositions and contents were measured in petal backgrounds and bases of PR, PO, LHD, MCJH, and HN during petal spot formation (Figure 2). Generally, flavones and flavonols were predominantly accumulated at the petal background of white and yellow flowers, including kaempferol (Km), apigenin (Ap), isorhamnetin (Is), luteolin (Lu), and chrysoeriol (Ch), which also occurred at petal bases at S1. Anthocyanins were abundantly accumulated from S2 and reached the highest peak at S4. For pink and purple flowers, the anthocyanin accumulation in petal spots was relatively higher than that of petal backgrounds. During dark-purple petal spot formation in PR and MCJH, peonidin 3, 5-*O*-glucoside (Pn3G5G), cyanidin 3, 5-*O*-glucoside (Cy3G5G), and cyanidin 3-*O*-glucoside (Cy3G) were the dominated anthocyanins and most abundant at S4. In addition, Pn3G5G and Cy3G were considered as the major anthocyanins and accumulated more and more abundantly during petal spots formation of LHD, whereas peonidin 3-*O*-glucoside (Pn3G) and Pn3G5G in HN. In contrast, flavonoids were accumulated similarly in petal bases with petal background in PO and PR (Figure 2).

### Identification and RNA *in situ* hybridization of *PrMYB5* in *P. rockii* petal

In our previous flower transcriptome database of *P. rockii* (Shi et al., 2017), an R2R3-MYB TF related to anthocyanin regulation named *PrMYB5* (Genbank accession number: c117848) was obtained. *PrMYB5* contained an 828 bp open reading frame (ORF), encoding a protein of 275 amino acids (Supplementary Figure 2A). PrMYB5 contained highly conserved R2 and R3 domains at the N-terminus, indicating that it belonged to the typical R2R3-MYB family. Meanwhile, phylogenetic analysis showed PrMYB5 in the same clade as petal spot formation regulated MYBs, including OgMYB1 and PeMYB11, full-red pigmentation regulatory PeMYB2, venation pattern regulatory PeMYB12, and anthocyanin-promoting MYBs, such as *Zea mays* C1 (ZmC1), ZmPL, PsMYB12L, and PsMYB114L (Supplementary Figure 2B). Furthermore, we detected the fluorescence PrMYB5-GFP (green fluorescent protein) fusion protein in the nucleus of *Allium cepa* cell protoplasts, suggesting that PrMYB5 is a nuclear-localized TF in regulating petal spot formation (Supplementary Figure 2C).

**Figure 3:**
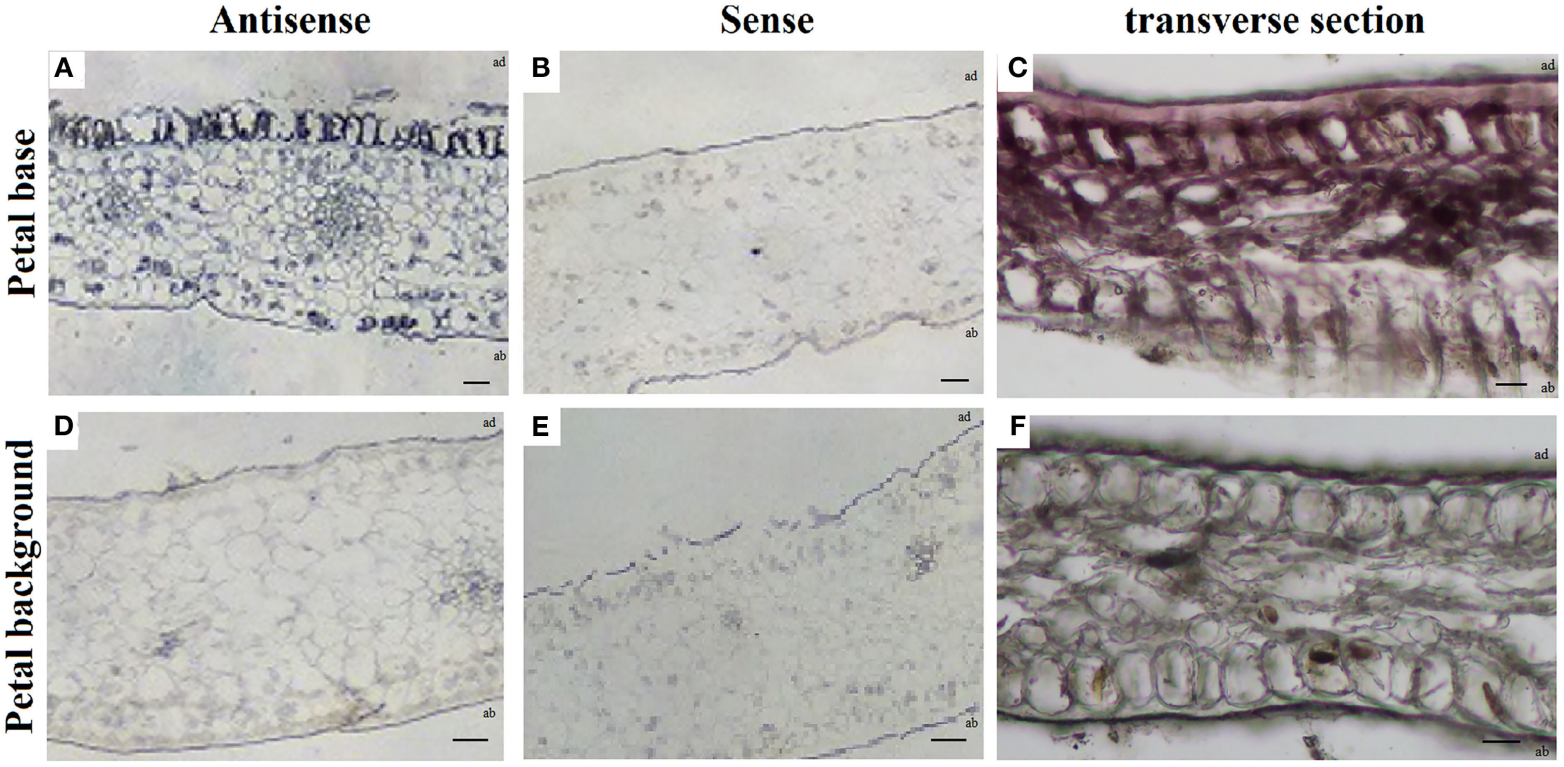
RNA *in situ* hybridization of *PrMYB5* transcripts in the stage 4 petals of *P. rockii*. **(A,B)** Transverse sections of the petal base of *P. rockii* were hybridized with antisense or sense RNA probes of *PrMYB5*. **(C)** Transverse section of the petal base of *P. rockii*. **(D,E)** Transverse sections of the petal background of *P. rockii* were hybridized with antisense or sense RNA probes of *PrMYB5*. **(F)** Transverse sections of the petal background of *P. rockii*. ab indicates Abaxial surface; ad indicates Adaxial surfaces. Bars = 100 mm.

RNA *in situ* hybridization in the petal background and petal base of *P. rockii* at S4 showed that PrMYB5 expressed more highly on the adaxial surface than on the abaxial surface of the petal base (Figure 3A). On the contrary, no expression of *PrMYB5* was detected in the epidermal cells of the petal background (Figure 3D). These results were consistent with the transverse section phenotypes (Figures 3C,F). As a negative control, hybridization with sense probes was conducted (Figures 3B,E). Therefore, *PrMYB5* showed distinct expression patterns in the petal background and petal base of *P. rockii*.

### Expression analyses of *PrMYB5* and structural genes in anthocyanin biosynthetic pathway

**Figure 4:**
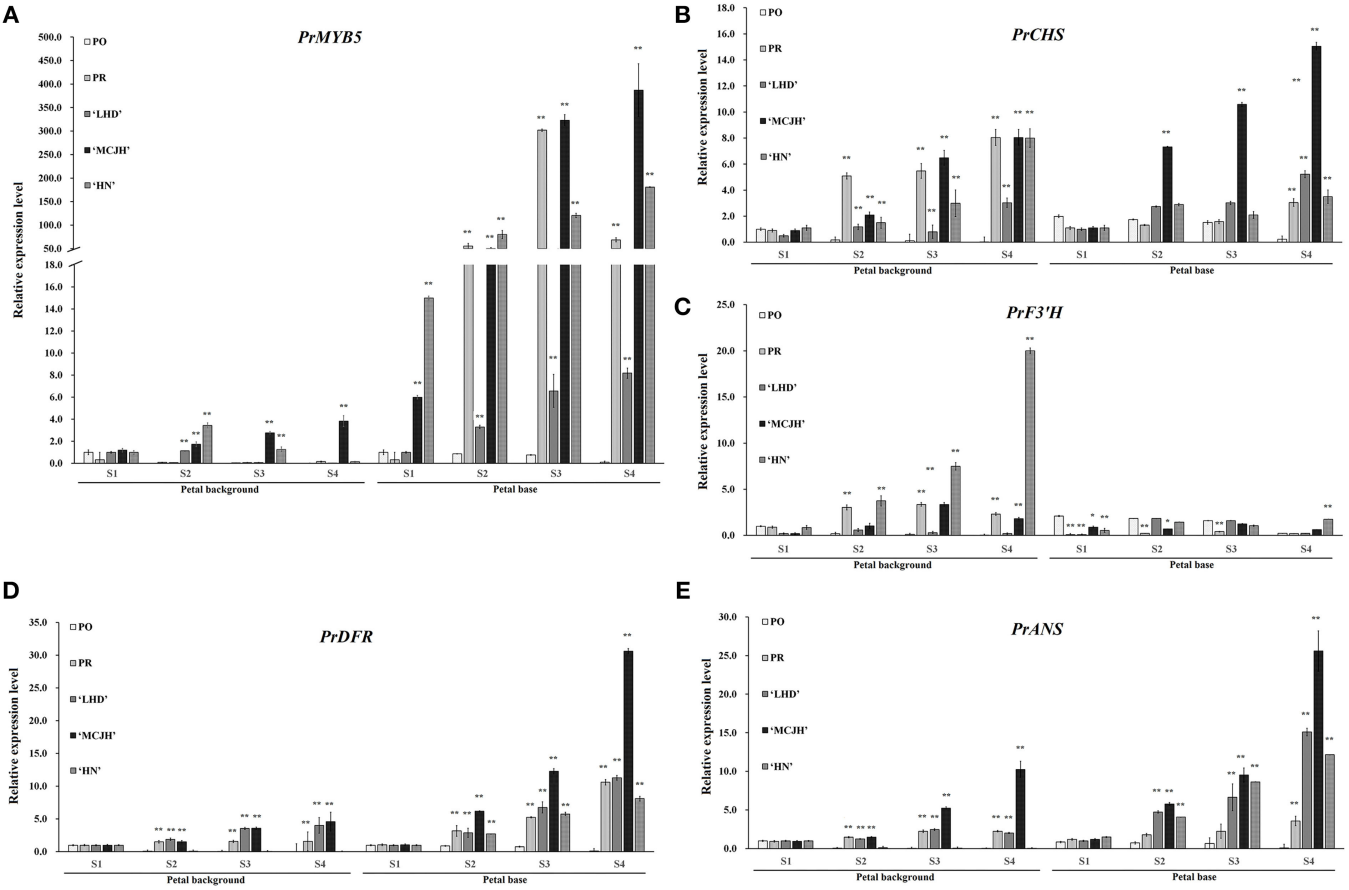
The expression profiles of *PrMYB5* and four related structural genes in *P. rockii* (PR), *P. ostii* (PO), and three tree peony cultivars “Lanhudie” (LHD), “Mochi Jinhui” (MCJH) and “High Noon” (HN) using qRT-PCR. The expression profiles of *PrMYB5* **(A)**, *PrCHS* **(B)**, *PrF3'H* **(C)**, *PrDFR* **(D)**, and *PrANS* **(E)** in all samples. S1–S4 represent four different blooming stages.

To further identify the function of *PrMYB5* associated with the petal spot formation, we analyzed the expression patterns of *PrMYB5* and four key structural genes (*PrCHS, PrF3*′*H, PrDFR*, and *PrANS*) that have been screened in our previous study (Shi et al., 2017). The expression levels of *PrMYB5* at the petal spot increased first and then decreased and peaked at S3 in PR, which were significantly higher than those at the petal background of *P. rockii* all the time. Compared to PR, *PrMYB5* is hardly expressed in all petals of PO (Figure 4A). In addition, the expression levels of *PrMYB5* in LHD, MCJH, and HN increased continuously from S1 to S4 (Figure 4A), and the transcript of *PrMYB5* at the petal base exhibited higher abundance than that at the petal background. In general, the expression level of *PrMYB5* increased dramatically with the spot formation in spotted species and cultivars. Especially, it is expressed in the highest abundance in the petal base of MCJH with darker petal spot (Figure 4A).

The transcripts of *PrCHS* and *PrF3'H* in petal background were higher than those at petal spot except for *PrCHS* in LHD and MCJH, whereas they displayed higher expression levels at petal base than petal background in PO (Figures 4B,C). However, *PrDFR* showed a gradually increasing expression trend at the petal bases of PR, LHD, MCJH, and HN from S1 to S4, which is similar with those of *PrANS* (Figures 4D,E). Especially, the expression levels of *PrDFR* were significantly more abundant at petal bases than those of *PrANS* from S1 to S4 in PR and MCJH. The interaction network based on flavonoid accumulation and gene expression profiles revealed that *PrMYB5* was positively correlated with *PrDFR* and *PrANS*, directly associating with Pn3G5G and Cy3G5G (Supplementary Figure 3).

### Overexpression of *PrMYB5* in tobacco

**Figure 5:**
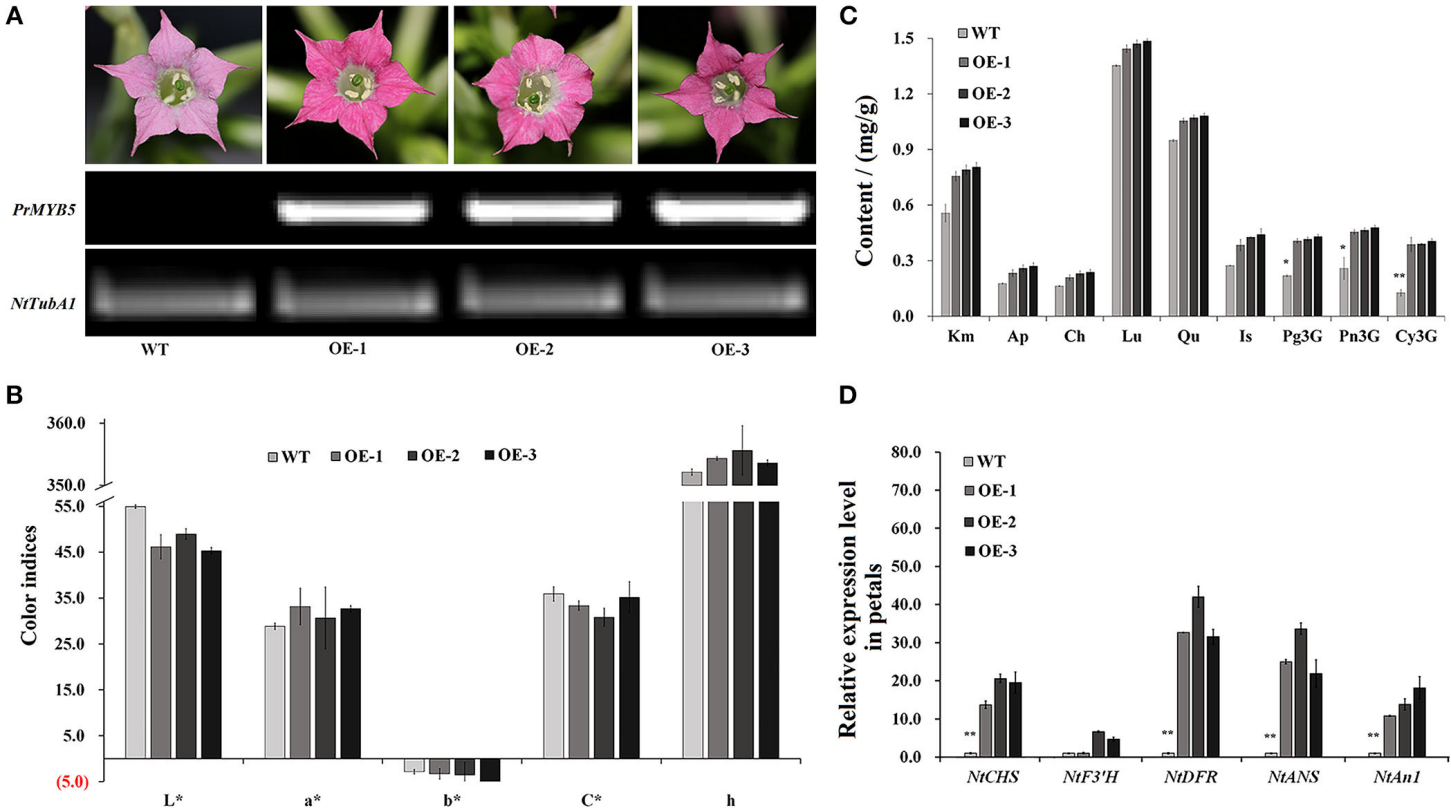
Overexpression of *PrMYB5* promotes the accumulation of anthocyanins in tobacco. **(A)** Flower phenotypes of OE-lines (OE-1, 2, and 3) and wild-type (WT) petals, and their corresponding *PrMYB5* transcription levels. *NtTubA1* was used as an endogenous control. **(B)** The color indices in OE-lines and wild-type petals. L* represents the lightness. a* represents the redness. b* represents the yellowness. C* represents chroma. *h* represents the hue angle. **(C)** The contents of pigments in OE-lines and wild-type petals. **(D)** Expression profiles of *PrMYB5* and structural genes in OE-lines petals by qRT-PCR. *Indicates significant differences at *P* < 0.05; **indicates relatively significant differences at *P* < 0.01.

To characterize *PrMYB5* function in anthocyanin biosynthesis, we overexpressed *PrMYB5* in tobacco plants and obtained three *PrMYB5* transgenic lines (OE-*PrMYB5* 1–3) (Figure 5). The petals of OE-*PrMYB5* displayed deeper red color compared with wild-type plants, while the sepals, stamens, pistils, and leaves of transgenic lines had no obvious difference (Figure 5A). All OE lines displayed higher transcripts than WT (Figure 5A). Consistently, L^*^ and b^*^ values of corollas in all OE-*PrMYB5* were observably lower than those in wild-type, whereas a^*^ value was higher in all OE-*PrMYB5* (Figure 5B). Furthermore, the corollas of three OE-*PrMYB5* lines accumulated more Pg3G, Pn3G5G, Pn3G, Cy3G5G, and Cy3G than those of wild-type (Figure 5C), but not obviously different from other flavonoid components (Figure 5C). The above results indicate that *PrMYB5* could significantly promote anthocyanin biosynthesis in tobacco.

The transcript of *PrMYB5* was 70-fold higher in petals of OE-*PrMYB5* than that in wild type, and those in leaves were also 26-fold more abundant (Figure 5D). Correspondingly, four structural genes were also upregulated in the petals of three OE-*PrMYB5* lines. Particularly, *NtDFR* and *NtANS* exhibited the most significant high transcripts in petals of three transgenic lines. The bHLH TF *NtAn1*, stimulating the anthocyanin biosynthesis in tobacco, upregulated in the petals of all three transgenic lines. In short, *PrMYB5* acted as a positive TF to activate anthocyanin accumulation in the petals.

### Transient silencing of *PrMYB5* in *P. rockii* petal

**Figure 6:**
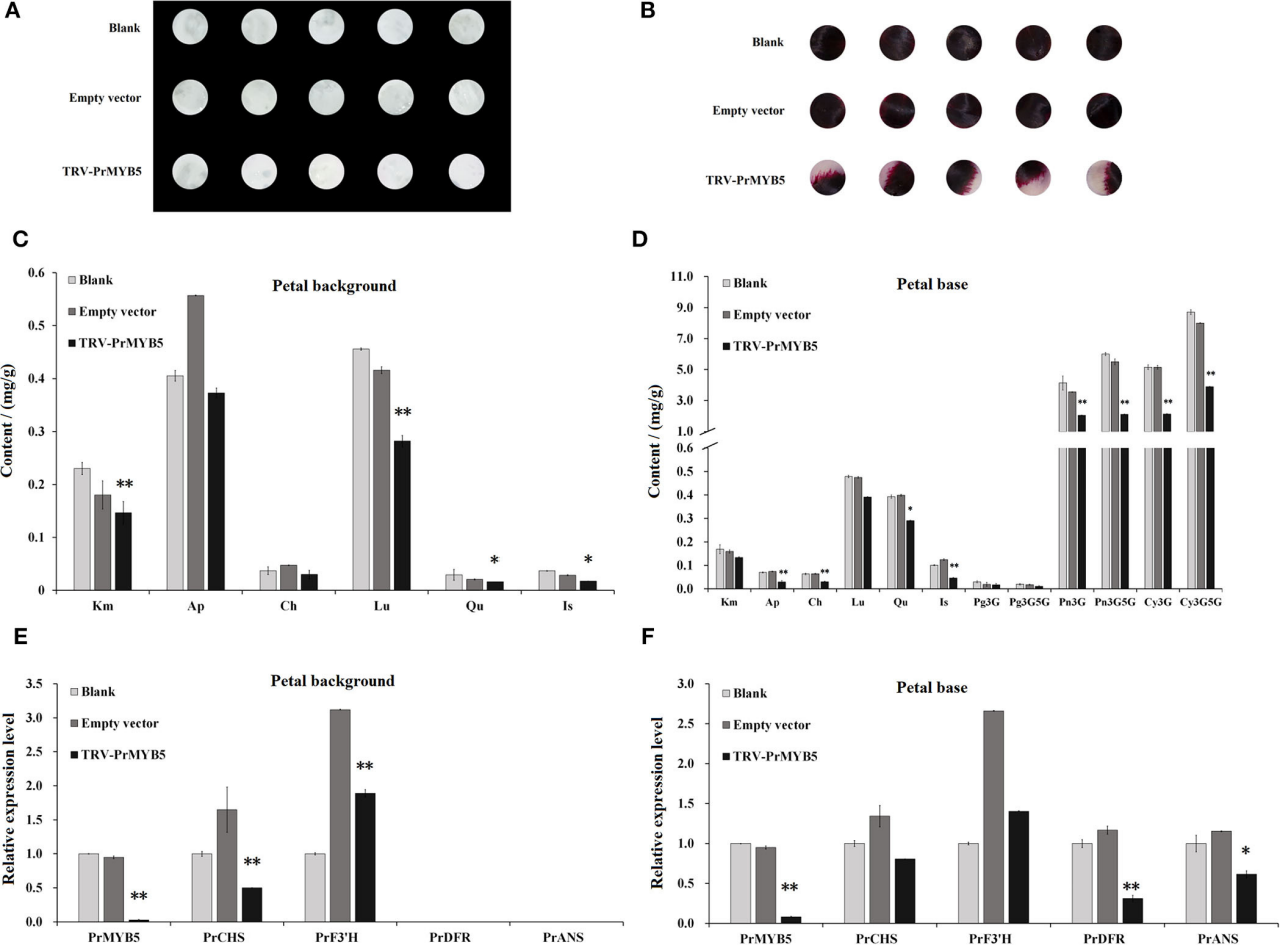
Transient silencing of *PrMYB5* in *P. rockii* petals using virus gene silencing (VIGS). Phenotypes of petal background disc **(A,B)**. The flavonoids accumulation in the petal background disc **(C)** and petal base disc **(D)**. The expression levels of *PrMYB5* and structural genes in petal background disc **(E)** and petal base disc **(F)**. *Indicates significant differences at *P* < 0.05; **indicates relatively significant differences at *P* < 0.01).

After VIGS were conducted in petal discs of *P. rockii*, the colors of all *PrMYB5-*silenced petal base discs were visibly lighter than those of the blank-control and empty vector control (Figures 6A,B). The changes of flavonoid contents were mainly concentrated in down-accumulation of Km and Lu in *PrMYB5*-silenced petal background discs (Figure 6C), and the contents of Cy3G, Cy3G5G, Pn3G, and Pn3G5G observably decreased in *PrMYB5*-silenced petal base discs (Figure 6D). Correspondingly, *PrMYB5* is down-regulated in *PrMYB5*-silenced petal background discs and base discs, accompanied by decreased expression levels of *PrCHS, PrDFR*, and *PrANS* (Figures 6E,F). It is noteworthy that *PrDFR* was significantly downregulated in *PrMYB5-*silenced petal base discs, indicating the direct positive regulatory relationship between *PrMYB5* and *PrDFR* contributing to petal spot formation.

### Regulatory relationship between *PrMYB5* and *PrANS / PrDFR* promoters

**Figure 7:**
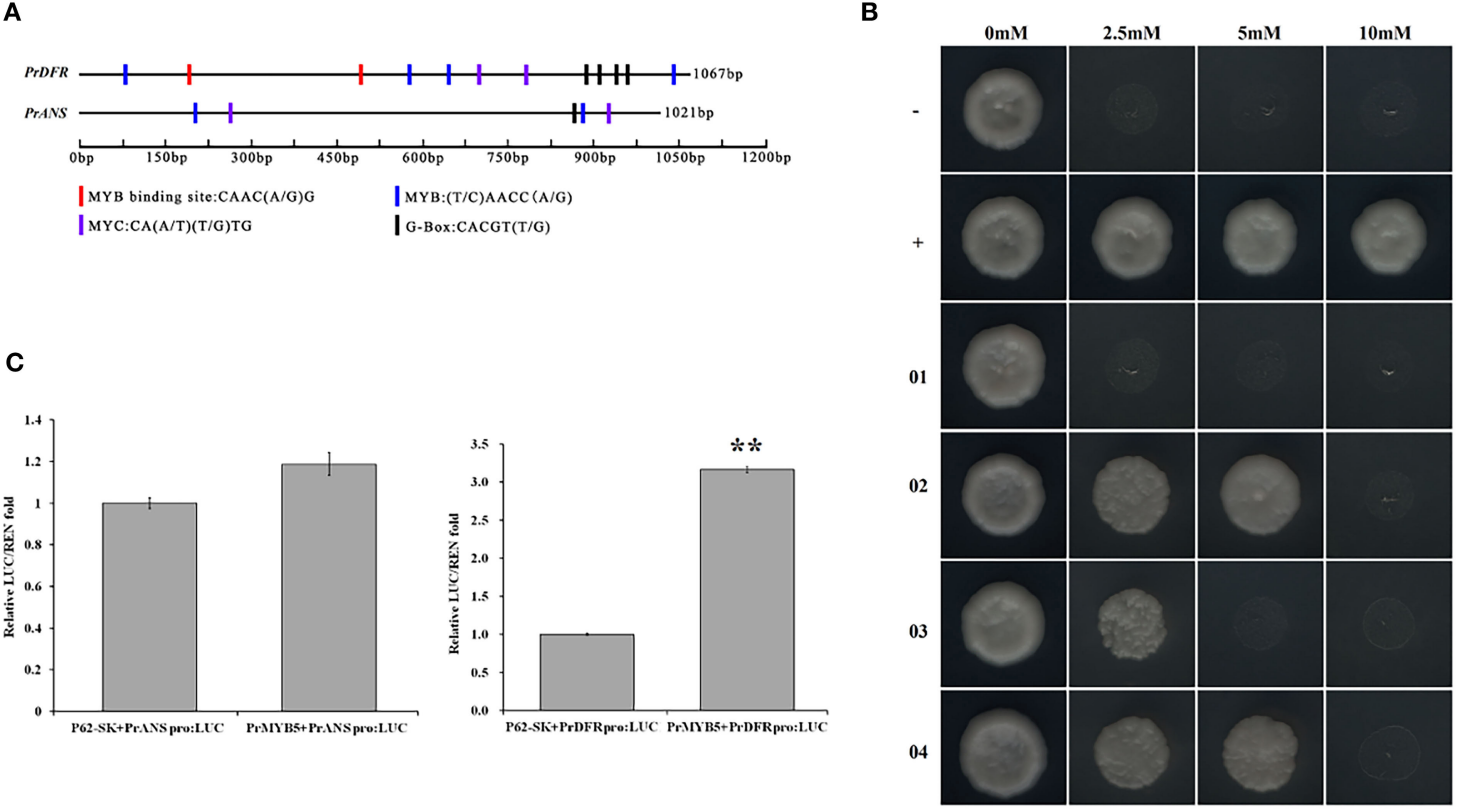
Transcriptional activity analysis of PrMYB5 against the promoters of *PrDFR* and *PrANS*. **(A)** Schematic overview of *PrDFR* and *PrANS* promoters. The lengths, MYB binding sites, MYB sites, MYC sites, and G-box sites are marked with black solid lines and colored rectangles, respectively. **(B)** Recombinant yeast on the SD medium without His, Leu, and Trp with 0 mM, 2.5 mM, 5 mM, and 10 mM indicate the SD medium without His, Leu, and Trp with 3-AT at concentrations of 0, 2.5, 5, and 10 mM, respectively. -, a negative control (pGADT7+p53::pHis2); +, a positive control (pGADT7::53+p53::pHis2); 01, pGADT7+*PrDFR*::pHis2; 02, *PrMYB5*::pGADT7+*PrDFR*::pHis2; 03, pGADT7+*PrANS*::pHis2; 04, *PrMYB5*::pGADT7+*PrANS*::pHis2. **(C)** Dual-luciferase assay in tobacco leaves. **Indicates relatively significant differences at *P* < 0.01.

To verify whether *PrMYB5 is* regulating *PrDFR* and *PrANS*, we cloned the promoter sequences of *PrDFR* (*PrDFRpro*) and *PrANS* (*PrANSpro*), with the length of 1,067 and 1,021 bp, respectively (Supplementary Figure 5). *PrDFRpro* included one MYB-binding site (5'-CAAC(A/G) G-3'), four MYB sites (5'-(T/C) AACC (A/G)-3'), two MYC sites (CA(A/T) (T/G) TG), and four G-box (5'-CACGT(T/G)-3'), while *PrANSpro* had two MYB sites, two MYC sites, and two G-box (5'-CACGT (T/G)-3') (Figure 7A), suggesting a potential regulatory relationship among *PrMYB5, PrDFR*, and *PrANS*.

To further verify whether *PrMYB5* activated *PrDFR* or *PrANS*, we first performed yeast one-hybrid (Y1H) assays (Figure 7B). Compared with the corresponding control groups, the yeast cells co-transformed with *PrMYB5*::pGADT7+*PrDFRpro*::pHIS2 and *PrMYB5*::pGADT7+*PrANSpro*::pHIS2 could grow normally on SD/-His/-Leu/-Trp selective medium with 2.5 mM 3-AT and 10 mM 3-AT, respectively (Figure 7B). Then, a dual-luciferase assay was conducted. The relative luciferase level of *PrMYB5*::SK+*PrDFR*::LUC was more than 3-folds than the control (SK+*PrDFR*::LUC), while no obvious enhancement was found in *PrMYB5*::SK+*PrANS*::LUC. Taken together, *PrMYB5* preferred directly activating the *PrDFR* promoter, promoting Cy- and Pn- anthocyanin at the petal base of *P. rockii* (Figure 7C).

## Discussion

The spot at the petal base of *P. rockii* mainly contributes to various spot patterns of tree peony (Wang et al., 2000; Shi et al., 2017). Pigmentation at the petal base begins much earlier than petal background pigmentation in tree peony (Figure 1, Supplementary Table 2), which is similar to those in snapdragons and lilies (Shang et al., 2011; Yamagishi et al., 2014). That is, petal spot pigmentation and petal background pigmentation may be independent.

Cy-based anthocyanins were the major cause of dark-purple spot, while Pn-based anthocyanins were for a vivid purple-red spot in tree peony (Shi et al., 2017; Gu et al., 2019). As expected, Cy3G, Cy3G5G, and Pn3G5G were the dominant anthocyanins in the dark-purple petal spot of PR and MCJH, whereas Pn3G5G and Cy3G in purplish red petal spots of LHD, and Pn3G5G and Pn3G in vivid red petal spots of HN (Figure 2). Whether the flower color is white, yellow, pink, or purplish red, the contents of anthocyanins were markedly higher in the petal spots, while flavones and flavonols accumulated at little different levels. Similar results have been found for petal spot formation in *Viola* × *wittrockiana* Gams. and *C. gracilis* (Martins et al., 2013; Li et al., 2014).

The biosynthesis of distinct Cy and Pn types of anthocyanins is regulated by structural genes and TFs (Suzuki et al., 2016). Among them, DFR and ANS catalyze the dihydroflavonols into the corresponding anthocyanins. Although they were expressed both in the red and purple colored petal areas, the expression levels of *PrDFR* in petal spot were observably different from those in the colored petal background, positively correlating with the accumulation of Pn3G5G and Cy3G5G (Figure 4; Supplementary Figure 3). Similar results in *C. gracilis, Oncidium* Gower Ramsey, and *Viola* × *wittrockiana*. Gams. showed that the different expression of *DFR* genes in petal spot activated anthocyanin production resulting in spot pattern (Chiou and Yeh, 2008; Martins et al., 2013; Li et al., 2014). Thus, the differences in anthocyanin accumulation between petal background and spot may be explained by the special spatial and temporal expression of *PrDFR*.

Among the TFs involved in anthocyanin biosynthesis, R2R3-MYB TFs play major roles in determining spot formation (Hsu et al., 2015). In this study, the R2R3-MYB anthocyanin regulator, *PrMYB5* was grouped in the same clade with OgMYB1, PeMYB11, ZmC1, ZmPL, and PsMYB12L (Supplementary Figure 2B), of which ZmC1, ZmPL, OgMYB1, and PeMYB11 belong to C1 subgroup of R2R3-MYBs, which is involved in upregulating anthocyanin biosynthesis (Jiang et al., 2004; Kranz et al., 2010; Stracke et al., 2010). In addition, *OgMYB1* was critical for the mosaic red pigmentation in the lip crest of *Oncidium* spp. (Chiou and Yeh, 2008), and *PeMYB11* was responsive to the red spots in the callus of the lip in *Phalaenopsis* spp. (Hsu et al., 2015). *PrMYB5* participating in the petal spot formation was further verified using *in situ* hybridization (Figure 3). In particular, *PrMYB5* showed a more abundant transcript at petal spots and upregulated gradually with the formation of spots at the petal base (Figure 4). In other plants, R2R3-MYBs in the C1 subgroup can regulate all structural genes in the flavonoid biosynthesis pathway, including *CHS, CHI, F3H, DFR*, and *ANS* (Chiu et al., 2010; Petroni and Tonelli, 2011; Liu et al., 2016; Schwinn et al., 2016), through binding MYB-binding sites or MYB sites' *cis*-elements with their promoters (Allan et al., 2008; Liu et al., 2016). Notably, a highly clear association existed among *PrMYB5, PrDFR*, and *PrANS* transcriptions during petal coloration and spot formation (Figure 4). In addition, Y1H and dual-luciferase assays revealed that *PrMYB5* had a significant activation effect on the *PrDFR* promoter. It has been reported that *McMYB10* can promote *McDFR1* expression and increase anthocyanins accumulation in *Malus* crabapple (Tian et al., 2017). *MdMYB10* and *MdMYB1* can promote the accumulation of anthocyanins in apple peels through binding to the *DFR* promoter (Fornale et al., 2014). Overexpression of *PrMYB5* in tobacco made petals appear deeper red, consisting with the increased Pg3G, Pn3G5G, Pn3G, Cy3G5G, and Cy3G levels and significantly upregulated *NtDFR* expression in petals of transgenic tobacco lines (Figures 5B–D). On the other hand, the reduction of Cy3G, Cy3G5G, Pn3G, and Pn3G5G caused by *PrMYB5* silencing in petal spots of *P. rockii* was consistent with the downregulation of *PrCHS, PrDFR*, and *PrANS*, particularly *PrDFR* (Figure 6), indicating that *PrMYB5* probably played a key role in petal spot formation of *P. rockii* through promoting *PrDFR* abundant expression at the petal base. Likewise, *LhMYB18* activated the promoter of the DFR gene in tobacco plants (Yamagishi, 2018), and *CgMYB1* activates *CgDFR2* to produce petal spots in *C. gracilis* (Martins et al., 2017).

## Conclusion

In summary, we compared the distinct pigmentation processes between petal bases and petal backgrounds of *P. rockii* and other tree peony cultivars, and further demonstrated that the petal spot formation was mainly attributed to the spatiotemporal transcription of *PrMYB5* activating *PrDFR* promoters, which was associated with the Cy and Pn type anthocyanin accumulation at the petal base. However, the complex and exact regulation mechanism of *PrMYB5* on petal spot formation requires to further research. These results not only provide a reference for further exploring the molecular mechanism of novel spot pigmentation patterns in *P. rockii*, but also lay the theoretical foundation for molecular breeding of novel spotted tree peony cultivars.

## Data availability statement

The datasets presented in this study can be found in online repositories. The names of the repository/repositories and accession number(s) can be found in the article/Supplementary material.

## Author contributions

QS and LL designed this research, performed data analysis, and wrote the manuscript. MY and SW performed the experiments and data analysis. XLu assisted in bioinformatics analyses. SL, YF, and XLi helped in the preparation of plant material. YZ provided some supervision of the study. All authors read and approved the final manuscript.

## Funding

This work was supported by the National Natural Science Foundation of China (Grant No. 31800599), the Natural Science Foundation of Shannxi Province, China (Grant No. 2017JQ3024), and the National Key R&D Program of China (Grant No. 2019YFD1001505).

## Conflict of interest

The authors declare that the research was conducted in the absence of any commercial or financial relationships that could be construed as a potential conflict of interest.

## Publisher's note

All claims expressed in this article are solely those of the authors and do not necessarily represent those of their affiliated organizations, or those of the publisher, the editors and the reviewers. Any product that may be evaluated in this article, or claim that may be made by its manufacturer, is not guaranteed or endorsed by the publisher.
