# Supplementary material for: PrMYB5 activates anthocyanin biosynthetic PrDFR to promote the distinct pigmentation pattern in the petal of Paeonia rockii
Source: Front Plant Sci. 2022 Aug 3;13:955590. doi: 10.3389/fpls.2022.955590 (PMC9382232; doi:10.3389/fpls.2022.955590)

**Supplementary Table S1 The specific primers used in this study.**

| Gene or vector names | Forward primer (5'-3') | Reverse primer (5'-3') | Restriction sites | Purpose |
| --- | --- | --- | --- | --- |
| *PrMYB5* | CACACAATCCACGCTCCAAAGGC | TGGAGTGAAGATGTGGGGGTGGG |  | 3'-RACE |
| *PrMYB5* | TGTGCTCTTTTGTCATTCTTATA | TGTGCTCTTTTGTCATTCTTAT |  | Full-length cDNA cloning |
| *PrMYB5* | AAAGGTACCTTATATATACTGCCAGGGTTG | AAAGTCGACCTGCTGCTGCTGCTGCTTATT |  | In-Fusion cloning |
| *PrCHS* | TATTGTCGGTGCTGATCCTG | TCACGCAAGTGTCCATCAAT |  | qRT-PCR |
| *PrF3’H* | ACCAGCAATTGGTCACATCA | GGTGATTGCGGATAATTCGT |  | qRT-PCR |
| *PrANS* | AACTAGCTCTTGGCGTGGAA | CAGAAAACTGCCCACGAAAT |  | qRT-PCR |
| *PrDFR* | CAGTACCCTCGGAGATTTCG | ATAGCCATGCCCTACACAGC |  | qRT-PCR |
| *PrMYB5* | ACCAGCAATCAATGACCATCTGTTACCCAGGAGC |  |  | RNA in Situ hybridization |
| *PrANS* |  | SP1: AAACCTCCCCTGCTTTCTTAACC |  | Promoters cloning |
|  |  | SP2: CATGGCCGCTTTCTTCAACTCCTC |  |  |
|  |  | SP3: GCCCTTCTTCTTTCTTCTCTTCCTC |  |  |
| *PrDFR* |  | SP1: CCCCCACAATAATTAATCACAC |  |  |
|  |  | SP2: GACGTAGCCATGTTCCAGAAG |  |  |
|  |  | SP3: TTTCAGCATCGCACTCGGTCA |  |  |
| pGADT7-*PrMYB5*-F | TGGCCATGGAGGCCAGTGAATTCATGGGAAGGGCTCCTTGTT | CTACGATTCATCTGCAGCTCGAGCTACTGCTGCTGCTGCTG | *Eco*R I+*Xho* I | Yeast one hybrid |
| pHIS2-*PrANS* | ACTCACTATAgggCgAATTCCgATTAgAgAggTAgCAAAggC | ATTCgCgAACgCgTgAgCTCTTTTgCAgCAACgTTTACTCTCT | *Eco*R I+*Sac* I |  |
| pHIS2-*PrDFR* | ACTCACTATAgggCgAATTCggTACgAggCAgTgATTCg | ATTCgCgAACgCgTgAgCTCTTgCTTTTgTTTTTTAACCACgATATgAg | *Eco*R I+*Sac* I |  |
| PrANS-0800 | GTACCGGGCCCCCCCTCGAGGTCGACCGATTAGAGAGGTAGCAAAG | GGCTGCAGGAATTCGATATCAAGCTTTTTTGCAGCAACGTTTACTC | *Sal I+Hind III* | Dual-luciferase assay |
| PrDFR-0800 | GTACCGGGCCCCCCCTCGAGGTCGACGGTACGAGGCAGTGATTCGAG | GGCTGCAGGAATTCGATATCAAGCTTTTGCTTTTGTTTTTTAACCACG | *Sal I+Hind III* |  |
| PrMYB5 -62 | GGCCGCTCTAGAACTAGTGGATCCATGGGAAGGGCTCCTTGTTG | ATCGATAAGCTTGATATCGAATTCCTACTGCTGCTGCTGCTGCT | *BamH I+EcoR I* |  |
| pCAMBIA1300-*PrMYB5* | AAAGGTACCTTATATATACTGCCAGGGTTG | AAAGTCGACCTGCTGCTGCTGCTGCTTATT | *Kpn* I+*Sal* I | Overexpression plasmid construction |
| TRV2-*PrMYB5* | GGGGTACCCCGAGAAGAACACCACCATCACC | CCGCTCGAGCGGCAACCCCCACATCTTCACTCC | *Kpn I+Xho I* | VIGS |
| *NtTubA1* | CTCCTATGCTCCTGTCATTTC | GGCGAGGATCACACTTAAC |  | QRT-PCR |
| *NtCHS* | TGACACCCACTTGGATAGTTTAG | CGACCTCTGGAATTGGATCAG |  |  |
| *NtF3'H* | AGGCTCAACACTTCTCGT | CATCAACTTTGGGCTTCT |  |  |
| *NtDFR* | AACCAACAGTCAGGGGAATG | TTGGACATCGACAGTTCCAG |  |  |
| *NtANS* | TGGCGTTGAAGCTCATACTG | GGAATTAGGCACACACTTTGC |  |  |
| *NtAn1* | GCAGCAGTGTTAGCTTGGATATG | AGCAGAACTCATTGAGGAAGACC |  |  |

**Supplementary Table S2 The flower color of the petal background and base during petal spot formation.**

| Stages | petal tissues | *Paeonia ostii* | *Paeonia rockii* | ‘Lanhudie' | ‘MochiJinhui' | ‘High Noon’ |
| --- | --- | --- | --- | --- | --- | --- |
|  |  | Color scheme | Color scheme | Color scheme | Color scheme | Color scheme |
| S1 | Petal background | Green | Green | Green | Green | Green |
|  | petal base | Green | Green | Green | Green | Green |
| S2 | Petal background | Pale yellow green | Light yellow green | Light yellow green | Light yellow green | Brilliant yellow green |
|  | petal base | Brilliant yellow green | Strong purplish pink | Deep purplish pink | Strong purplish red | Vivid purplish red |
| S3 | Petal background | Pale yellow green | Light yellow green | Pale yellow green | Light yellow green | Brilliant yellow green |
|  | petal base | Brilliant yellow green | Strong purplish pink | Strong purplish red | Deep purplish red | Vivid red |
| S4 | Petal background | White | White | Very pale purple | Strong reddish purple | Light greenish yellow |
|  | petal base | White | Dark purple | Moderate purplish red | Dark purple | Strong red |

S1-S4 represents four stages.

**Supplementary** **Figure S1 The color indices of *Paeonia rockii, P. ostii, P. suffruticosa* ‘Lanhudie’, ‘Mochi Jinhui’, and ‘High Noon’ at four different opening stages.** (A) *P. rockii*; (B) *P. ostii*; (C) ‘Lanhudie’; (D) ‘Mochi Jinhui’; (E) ‘High Noon’. S1-S4 represents four different blooming stages. *L** represents the lightness; the color becomes brighter as the value increases. *a** represents the redness. *b** represents the yellowness. *C** represents chroma; the color saturation increases as the value rises. *h* represents the hue angle; 0° (360°) is defined as purple–red, 90° is defined as yellow, 180° as blue–green, and 270° as blue.


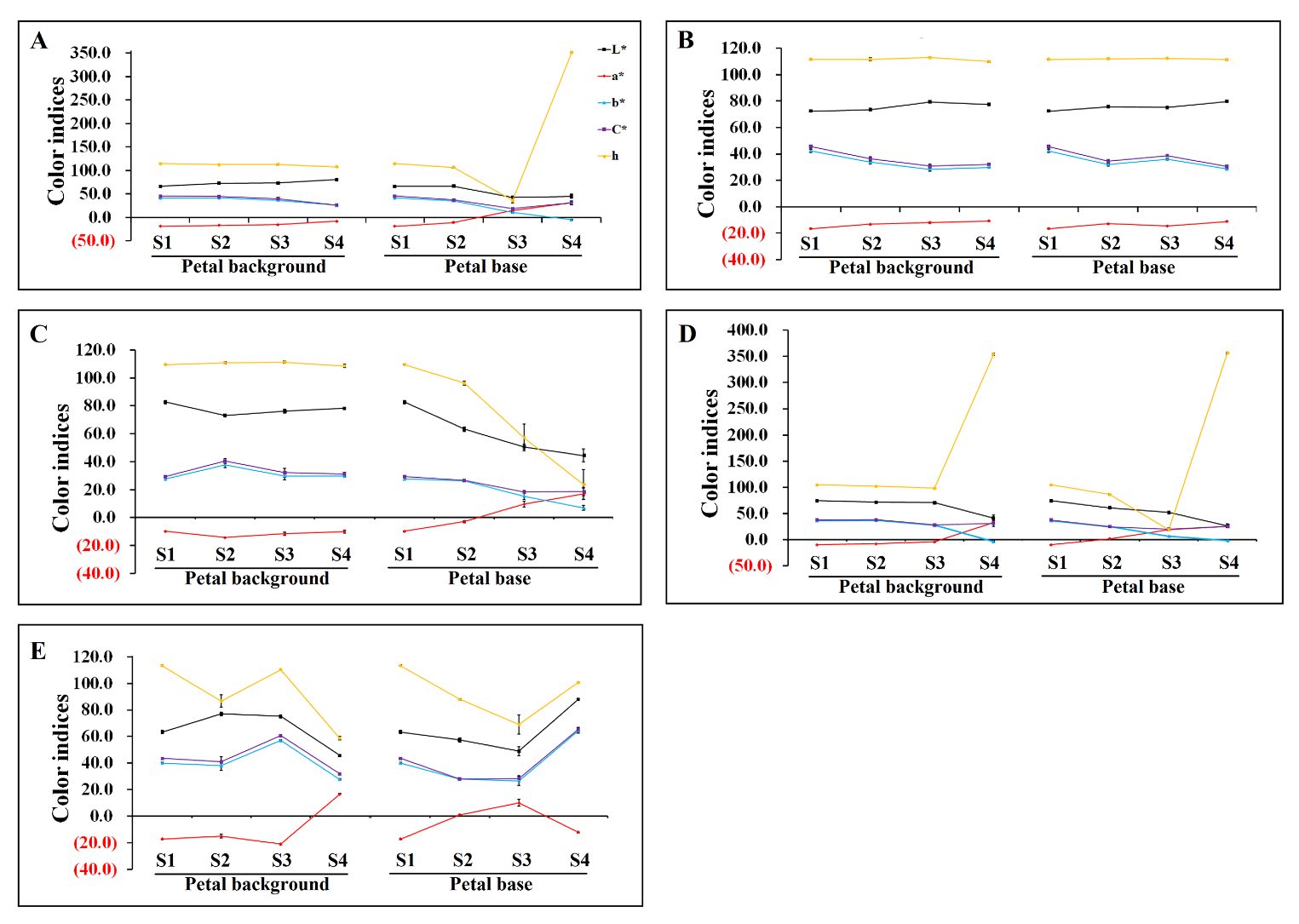


**Supplementary Figure S2** **Multiple alignment, phylogenetic tree analysis and subcellular localization of PrMYB5. (A) Multiple alignment of PrMYB5 amino acid sequence with other MYBs involved in anthocyanin biosynthesis and petal spot formation from other species.** Solid underlines denote R2 and R3 conserved domains at the N-terminus. **of PrMYB5. (B) Phylogenetic tree of PrMYB5 and several anthocyanin biosynthesis and petal spot regulating MYBs from other species using MEGA 6.0 software.** The phylogenetic tree was constructed with 1000 bootstrap value using the neighbor-joining method. PrMYB5 is represented by a black dot. The GenBank accession numbers of MYBs are as follows: AtPAP1 (NP_176057), AtPAP2 (NP_176813.1), AmROSEA1 (ABB83826), AmROSEA2 (ABB83827), AmVENOSA (ABB83828), CgMYB1 (AOW41206), GhMYB10 (CAD87010.1), LhMYB6 (BAJ05399), LhMYB12 (BAJ05398), LhMYB12-Lat (QYI40132), LhMYB19LONG (BCD56237), OgMYB1 (ABS58501), PhAN2 (AF146702_1), VvMYBA1 (XP_010664911.1), VvMYBA2 (BAA23339.1), PeMYB2 (AIS35919), PeMYB11 (AIS35928), PeMYB12 (AIS35929), PsMYB114L (QBK15079.1), PsMYB12L (QBK15080), PsMYB58 (AMW36067), PsMYB57 (QIG55740), ZmC1 (P10290.1), ZmPL (AAB67720). **(C)** Subcellular localization of PrMYB5 in onion epidermal cells. Bars, 10μm.


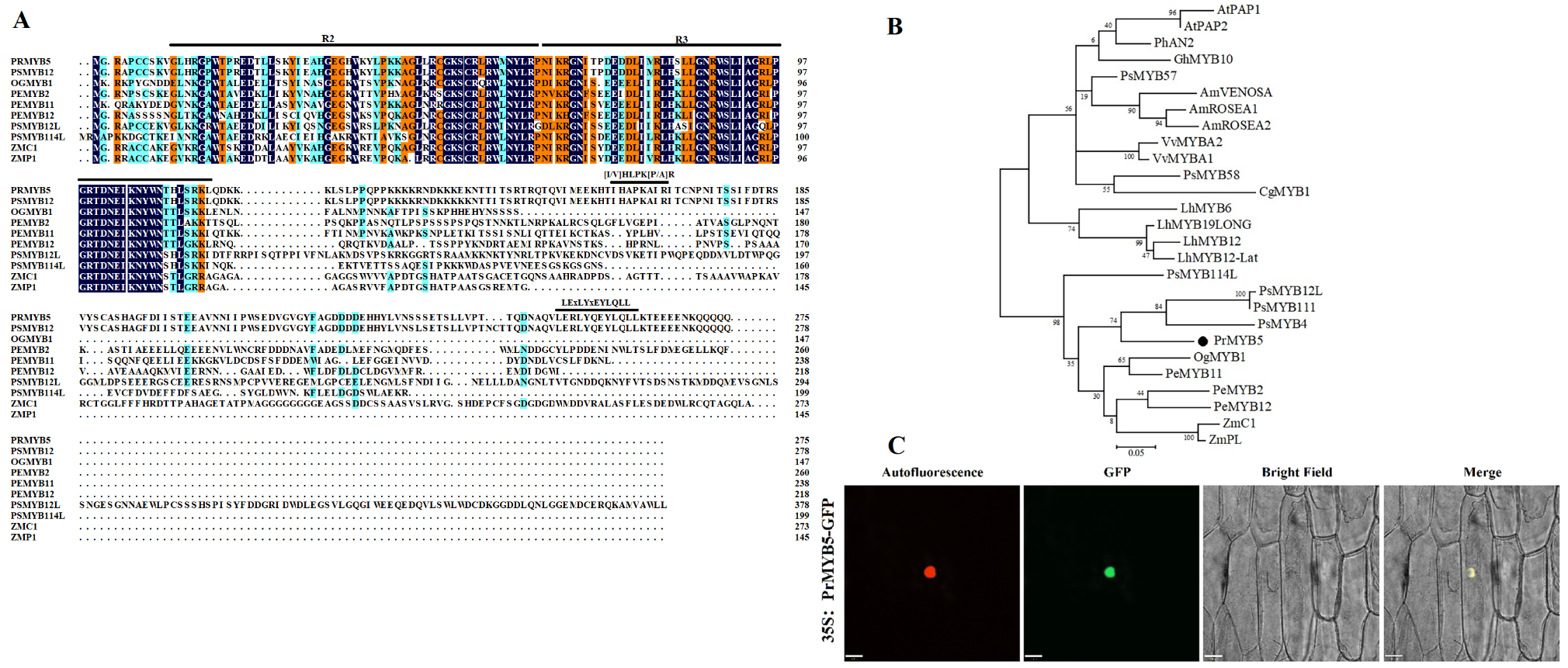


**Supplementary** **Figure S3 An interaction networkbetween transcript levels of genes and contents of flavonoids was constructed using the R package (https:// www. r- project.org) with a coefficient of R ≥ 0.5 or R ≤ -0.5 and Cytoscape (v.3.1.0).**


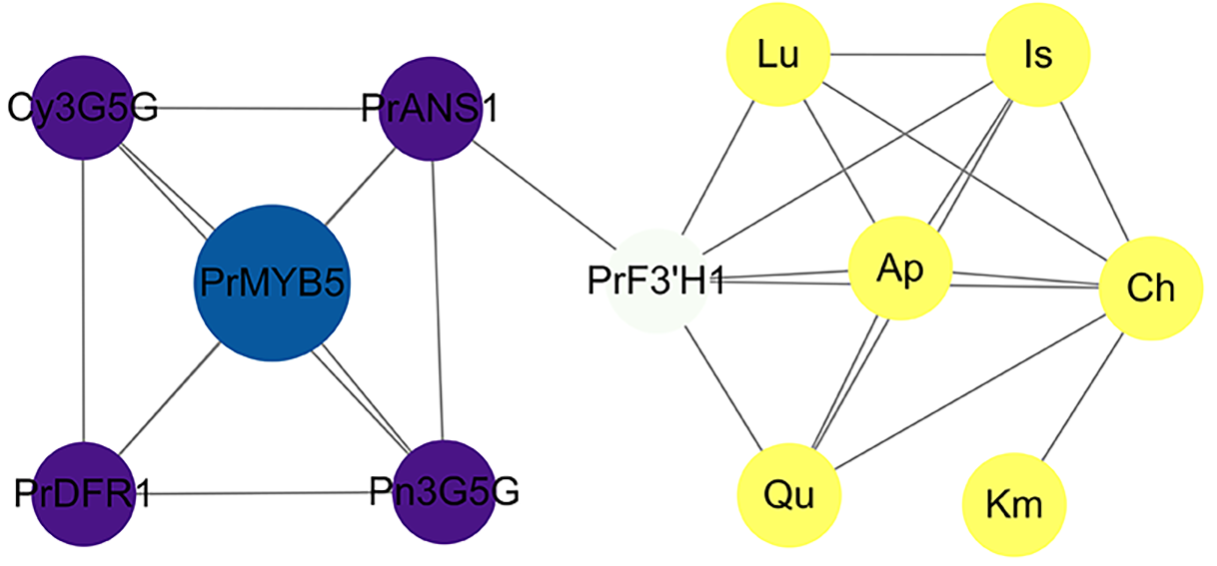


**Supplementary Figure S4 Promoter region sequence and its predicted cis-acting elements of *PrDFR* and *PrANS* from *P. rockii*. (A)** *PrDFR*; **(B)***PrANS*.

Gray: The main cis-acting elements predicted by PlantCARE; Overstriking: Marked +1, the upstream of which are negative positions.


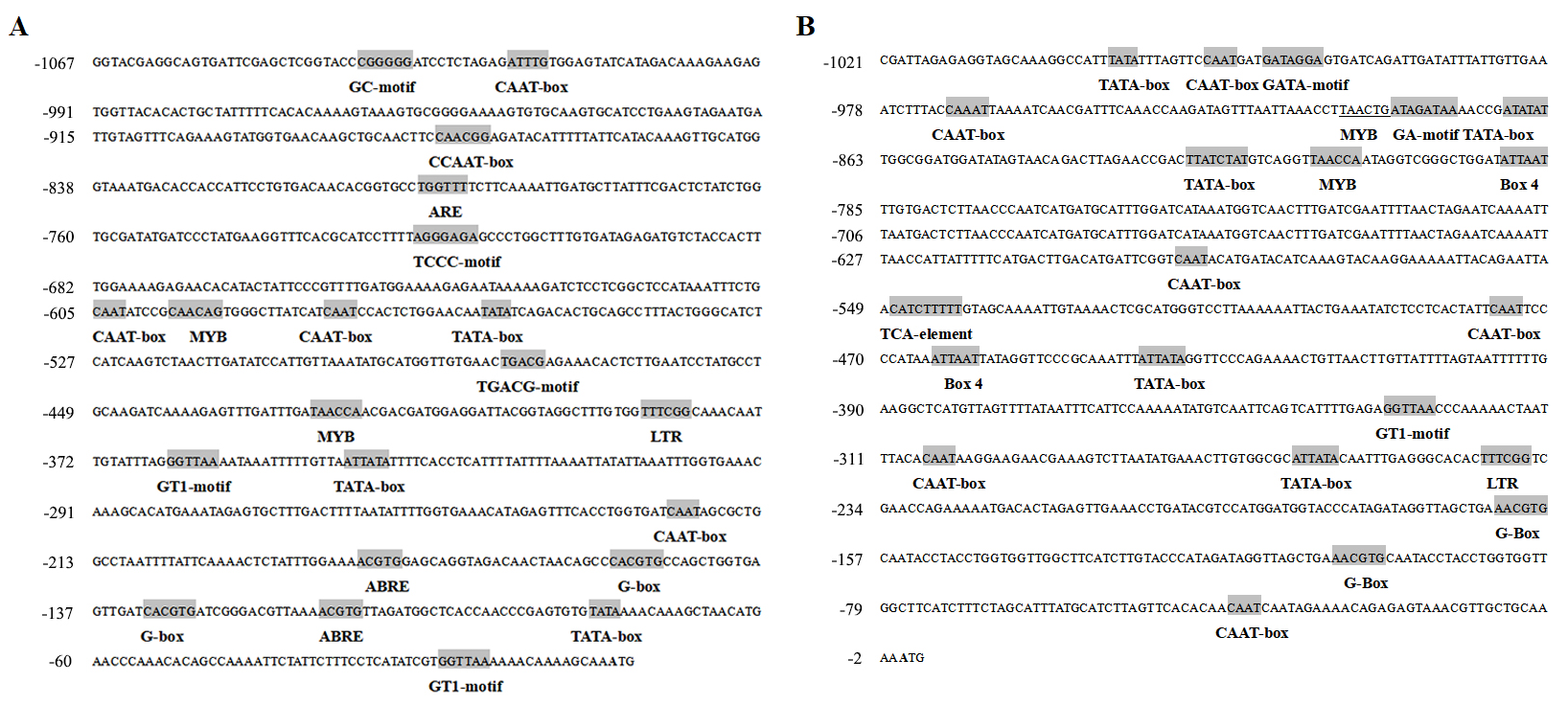

Supplement: Supplementary file 1 [file Data_Sheet_1.DOCX]
